# Refining the implementation research logic model: a citation analysis, user survey, and scoping review protocol

**DOI:** 10.3389/frhs.2024.1490764

**Published:** 2024-10-24

**Authors:** James L. Merle, Elizabeth A. Sloss, Olutobi A. Sanuade, Rebecca Lengnick-Hall, Rosemary Meza, Caitlin Golden, Rebecca G. Simmons, Alicia Velazquez, Jennie L. Hill, Paul A. Estabrooks, Mary M. McFarland, Miriam R. Rafferty, Dennis H. Li, Justin D. Smith

**Affiliations:** ^1^ Department of Population Health Sciences, Division of Health System Innovation and Research, Spencer Fox Eccles School of Medicine at The University of Utah, Salt Lake City, UT, United States; ^2^ College of Nursing, University of Utah, Salt Lake City, UT, United States; ^3^ Brown School, Washington University in St. Louis, St. Louis, MO, United States; ^4^ Kaiser Permanente Washington Health Research Institute, Seattle, WA, United States; ^5^ Department of Health & Kinesiology, College of Health, University of Utah, Salt Lake City, UT, United States; ^6^ Department of Obstetrics and Gynecology, Spencer Fox Eccles School of Medicine at The University of Utah, Salt Lake City, UT, United States; ^7^ Spencer S. Eccles Health Sciences Library, University of Utah, Salt Lake City, UT, United States; ^8^ Shirley Ryan AbilityLab & Departments of Physical Medicine and Rehabilitation and Psychiatry and Behavioral Science, Northwestern University Feinberg School of Medicine, Chicago, IL, United States; ^9^ Department of Psychiatry and Behavioral Sciences and Institute for Sexual and Gender Minority Health and Wellbeing, Northwestern University Feinberg School of Medicine, Chicago, IL, United States

**Keywords:** scoping review protocol, implementation research logic model, implementation science, citation analysis, survey

## Abstract

**Introduction:**

The Implementation Research Logic Model (IRLM) aids users in combining, organizing, and specifying the relationships between important constructs in implementation research. The goal of the IRLM is to improve the rigor, reproducibility, and transparency of implementation research projects. The article describing the IRLM was published September 25, 2020 (*Implement Sci*, Vol 15); it has since been highly cited and included as a required element in multiple funding opportunity announcements from federal agencies. The proliferation of IRLM use across dissemination and implementation research projects and practice provides an excellent opportunity to examine applications across a variety of different contexts. This protocol will result in a description of the impact of the IRLM on the field of dissemination and implementation science and guidance on refinements to the IRLM to increase its utility and impact through (1) a citation analysis, (2) a scoping review, and (3) user surveys and interviews.

**Methods and analysis:**

This protocol follows the Preferred Reporting Items for Systematic Reviews and Meta-Analyses extension for scoping review reporting guidelines (PRISMA-ScR). We plan to conduct a citation search and analysis of the Smith et al. 2020 article and a scoping review. The review search will be conducted in Medline, Embase, CINAHL Complete, Cochrane Library, APA PsycINFO4, ProQuest Dissertations & Theses Global, Scopus and Web of Science Core Collection., and grey literature will be searched to identify studies that use alternative logic models for implementation research. A survey will be developed from the findings of the scoping review and administered to individuals who used the IRLM. Semi-structured interviews will then be conducted with a sample of survey respondents to provide an opportunity for sequential mixed-methods analysis to achieve a deeper understanding of needed IRLM refinements and recommendations.

**Ethics and dissemination:**

Ethics approval for the scoping review and citation analysis is not applicable as only data from published literature will be used and no original data will be collected. For the survey, IRB will be completed once items are developed from the results of the scoping review and citation analysis. Results will be disseminated through peer-reviewed publications, conference presentations, and via online tools.

**Registration details:**

This protocol was registered with OSF, https://osf.io/y94bj (1).

## 1 Introduction

Implementation research often requires the use of multiple conceptual frameworks to guide different aspects of a project. Determinant frameworks are used to characterize the implementation context (2). Strategies, often across multiple levels within systems (e.g., policy, inner setting, outer setting, individual) aligned with prominent contextual factors (barriers and facilitators to implementation), are used to support adoption and delivery of evidence-based innovations (3, 4). These strategies operate through mechanisms of action to achieve implementation outcomes (5). The Implementation Research Logic Model (IRLM) (6) is a tool that aids users in combining and organizing these key elements and in specifying the relationships between them. The goal of the IRLM is to improve the rigor, reproducibility, and transparency of implementation research projects. The primary IRLM paper was published September 20, 2020, with several national presentations given prior to publication of the article. Since then, it has been well cited and has been required in Notices of Funding Opportunity issued by the National Institutes of Health, the Centers for Disease Control and Prevention, and the Agency of Healthcare Research and Quality (e.g., https://grants.nih.gov/grants/guide/notice-files/not-ai-23-070.html).

Given the required inclusion in grant applications, there is a need for instructional resources for IRLM users. Tools such as fillable templates have been made available as Supplementary Material to the article and through websites. More recently, the HIV Implementation Science Coordination Initiative (ISCI), the national coordinating center for the NIH's EHE grant program, produced an interactive online IRLM design tool (irlm.isgmh.northwestern.edu). However, users of these resources reported still needing a high level of implementation science expertise when first learning about the IRLM, populating its components, and drawing connections between elements. They expressed a need for additional guidance on using the IRLM. Specifically, they wanted to know when and where to start, what level of detail to include, how often to update, and how to effectively use with community partners without overwhelming them.

It is common as scientific fields mature for models and frameworks to undergo refinement and updates to reflect shifting perspectives and the accumulation of data. In recent years, three often-used implementation science frameworks have published updated versions. Developers and investigators using the RE-AIM planning and evaluation framework published multiple updates, systematic reviews, and a 20-year retrospective article in 2019 (7) that described the application and evolution of RE-AIM and discussed challenges with use and lessons learned (8, 9). Across these publications, the authors provided ongoing refinements and recommendations for the RE-AIM framework that included pragmatic use, cost, and adaptation considerations. The Exploration, Preparation, Implementation, and Sustainment (EPIS) process and determinants model published an update, also in 2019 (10) based on a systematic review of the literature that examined and described the research application of EPIS to understand how the framework was being used. Authors identified 49 unique projects and extracted data related to key aspects of EPIS application (e.g., study design, levels of data collection and analysis, comprehensiveness and depth of EPIS use within the project). In response to their findings, the authors refined definitions among factors and provided additional recommendations for EPIS use. Lastly, the Consolidated Framework for Implementation Research (CFIR) published the Updated CFIR in 2022 (11), as well as a companion outcomes addendum (12), following a systematic review of the literature and a user survey. Their purpose was to elicit feedback from experienced users to inform updates to the framework. This process led to significant changes to the scope of CFIR as well as changes to the framework's organization of domains and constructs. Several changes in the updated versions of these frameworks had been well-known challenges and sources of confusion in the field, which had been allowed to persist for several years, suggesting a need for processes to examine use in the field to inform an update on more frequent and routine intervals.

In this project, we aim to understand how the IRLM has been used by conducting (a) a citation analysis of the original IRLM paper, (b) a scoping review of peer-reviewed and grey literature, and (c) a user survey with follow-up interviews. Five sources were searched for existing reviews or protocols on July 15, 2024: PubMed, pubmed.gov; Epistemonikos, www.epistemonikos.org; PROSPERO, www.crd.york.ac.uk/PROSPERO; Open Science Framework, osf.io and Preprint Citation Index (Web of Science). No results were on topic to our research question.

Together, these methods will allow us to identify and evaluate IRLMs in the literature, summarize how and why the IRLM is being cited, and gather direct user feedback to offer timely recommendations and improvements for its use. The overall goals of this project are to improve the usability, accessibility, and theory of the IRLM and to generate resources to guide users.

## 2 Methods and analysis

### 2.1 Overview

The citation analysis will map the landscape and assess the scientific spread and scholarly impact of the IRLM. The scoping review search strategy and methods will allow us to examine IRLMs that have been used in implementation projects and to identify grey literature and additional projects not captured by peer-reviewed journals. Our findings from the first two phases will inform the development of a user survey and semi-structured interview guide to obtain feedback on the IRLM (e.g., use, utility, clarification needs) to inform refinements and resources to support users.

### 2.2 Citation analysis

To identify citing articles, a direct, forward citation search (13) within Google Scholar will be conducted. Human coders will use content-based citation analysis (14), which includes both semantic and syntactic analysis of citing articles, such as where in the manuscript the citation appears and the context or purpose of the citation. We will extract reasons for the citation, and any shortcomings or refinements to the IRLM that are discussed. We will use this method to identify IRLMs published as figures or as Supplementary Material and to request additional IRLMs from authors who indicated using the IRLM but did not include it in the published article.

### 2.3 Scoping review

We will conduct our scoping review with guidance from the JBI Manual for Evidence Synthesis (15, 16). Utilizing the framework as outlined by Arksey and O'Malley, we will conduct our scoping review with Arksey's five stages: (1) identifying the research question, (2) identifying relevant studies, (3) study selection, (4) charting the data and (5) collating, summarizing and reporting the results (17). Their optional sixth stage for stakeholder engagement will frame our user survey. For transparency and reproducibility, we will adhere to the Preferred Reporting Items for Systematic Reviews and Meta-Analyses extension for scoping review reporting guidelines (PRISMA-ScR) (18, 19).

The aim of our scoping review is to provide evidence on how the IRLM, or logic modeling and implementation research, has been used to plan or execute projects worldwide. We will use the *Concept* and *Context* of the JBI mnemonic of PCC for *Participants, Concept, Context* to frame our research question.

• Research question: How has the IRLM, or logic modeling and implementation research, been used to plan or implement research projects within and outside of healthcare settings worldwide?
• Concept: uses of the IRLM, or logic modeling and implementation research used to plan or implement research projects
• Context: Worldwide use or application in any settings since 2018, which was when the first formal presentation of the IRLM was given the Academy Health 11th Annual Conference on the Science of Dissemination and Implementation (20).

An information specialist (MMM) will develop the search strategies using a combination of keywords and database subject headings for the primary databases, Medline and Embase, from sentinel studies and team feedback, then translate the strategy to the other selected databases. Library colleagues will peer review the strategy according to PRESS guidelines (21). Citation management and duplicate detection and removal will be accomplished with EndNote (Clarivate Analytics).

Databases will include Medline (Ovid) 1946–2024, Embase (Elsevier) 1974–2024, CINAHL Complete (Ebscohost) 1937–2024, Cochrane Library (Wiley) 1898–2024 including CENTRAL (wiley.com) 1898–2024, APA PsycINFO (Ebscohost) 1872–2024, ProQuest Dissertations & Theses Global 1861–2024, Scopus (Elsevier) 1970–2024 and Web of Science Core Collection (Clarivate) 1900–2024. A date limit from 2018 will be applied. References of included studies will be checked for relevant inclusion. The full database search strategy including search terms are included as a Supplementary Material.

We also plan to conduct a grey literature search (22) to identify additional IRLMs and projects not captured through the search of peer-reviewed journals. A grey literature search will be conducted (a) dissemination and implementation-related conference proceedings between 2018 and 2024 and (b) consultations with corresponding authors or content experts on published papers. Conference proceedings will be reviewed across three societies: (1) AcademyHealth: Annual Conference on the Science of Dissemination and Implementation, (2) Global Implementation Society: Global Implementation Conference, and (3) Society for Implementation Research Collaborative (SIRC): Biannual Conference.

### 2.4 Study selection and eligibility criteria

To select studies, we will include records that (a) were conducted during or after the year 2018, (b) are written in English, and (c) are related to logic modeling and implementation research. Non-English studies will be excluded at full text review as no translation funding is available.

Inclusion criteria:

Concept: Uses the IRLM or implementation-related logic modeling.

Context: Worldwide use or application in any settings since 2018.

Exclusion criteria: Study is not implementation research per definition in Smith et al. (23).

A team of trained readers will screen titles and abstracts, then review full text using Covidence (24). Two readers from a pool of seven will review each record, and discrepancies will be reconciled by senior team members experienced with implementation science frameworks, theories, and methods. After all articles are screened, a random sample audit of 100 articles will be re-screened with a 5% threshold for misclassification. At full text review, reviewers will record the *a priori* reason for exclusion of each study.

### 2.5 Quality assessment

In compliance with scoping review methodology, no quality assessment of included studies will be conducted, as our goal is to rapidly map the literature.

### 2.6 Review team characteristics and training

Our review team will consist of individuals who have doctoral- and/or postdoctoral-level training in implementation science. We will utilize a stratified leadership model, wherein senior-level researchers (including the IRLM co-developers; JDS, DHL, MRR, PAE, JLH) will oversee early-career faculty & postdoctoral researchers (JLM, ES, OAS, RLH, RM, CG, RGS). Ongoing weekly virtual meetings will be held to share concepts and processes and adjust timelines according to individual and collective progress. These meetings will also provide space and opportunity to raise questions about the coding or concepts, contribute to decision making and codebook refinement, and prevent coder drift.

### 2.7 Data extraction and coding

Study- and sample-level data from the corpus of articles and studies identified by the citation analysis and the scoping review will be extracted using Covidence (24). Included articles from both methods of identification will be categorized into two groups: for articles that include an IRLM, we will extract details to categorize and evaluate its comprehensiveness; for articles that do not include an IRLM but cite the article, we will characterize the reason(s) for citation. Study-level variables (e.g., author, title, year published, study setting) and any recommendations for improving the IRLM will be extracted from each included study. If a novel format for the IRLM figure was included, the following categories will be extracted: (a) the IRLM template they used; (b) the study design phase(s) in which the IRLM was completed; (c) the comprehensiveness of data elements included in the IRLM; (d) which frameworks or taxonomies were used within the IRLM; and (e) any shortcomings and recommendations for improving the IRLM discussed. If no IRLM figure is included, studies will be coded for (a) citation purpose and location of citation in the article and (b) any noted benefits, deficiencies, or recommendations for improving the IRLM. For studies included in the scoping review that neither include an IRLM nor cite the IRLM paper, we will extract information, such as which logic model was used (e.g., program evaluation logic model). Each study will be double coded with a consensus round conducted by the senior research team. Extraction training will involve synchronous training sessions followed by assigned practice articles.

An initial draft of an extraction codebook was developed, informed by the original IRLM paper and after reviewing several articles included in the citation search. Iterative codebook development and refinement was conducted by authors JLM, JDS, DHL, and RGS by piloting 12 randomly chosen articles and conducting practice coding. Six articles were assigned to each coder, with coders meeting on multiple occasions to compare codes, discuss, and make refinements. The final codebook with operational definitions is included as a Supplementary Material.

### 2.8 Analysis of the evidence

Summary information for each extraction category will be analyzed and presented in planned manuscripts according to the categories detailed above. Additional post-hoc qualitative analyses are planned to further elucidate themes within and across implementation settings.

### 2.9 Presentation of results

Results will be presented using a combination of tabular, graphical, and narrative forms.

### 2.10 Dissemination of results

Results of our scoping review will be submitted to a peer-reviewed journal for publication and disseminated at conference presentations.

### 2.11 Mixed-methods user study for IRLM feedback

In addition to the citation analysis and scoping review, we plan to conduct a sequential mixed-methods user study sampling those who have used the IRLM in implementation projects. Specifically, we will administer an online survey to all users who have cited the IRLM in indexed publications and grey literature and indicated using it in a project. We will also ask participants to share whether they included the IRLM in a grant proposal. Survey participants will be asked to provide demographic information and indicate if they are willing to participate in follow-up, semi-structured interviews. We will also ask respondents to share contact information for anyone who has attempted to use the IRLM and/or not used it successfully; we will reach out to anyone identified to see if they are willing to participate in an interview. Interviews will take place via a secure video conferencing platform. At the conclusion of survey and interview data collection, we will convene an expert panel which will function like a focus group to discuss overall feedback and identify recommendations for improving the IRLM.

#### 2.11.1 Sampling strategy

Invitations for the survey will be sent to the corresponding authors via email with an embedded link to the survey. Should we receive no response after 14 days, including a reminder email after 7 days, we will email the senior author with the survey link. Unique links will be used to ensure only a single survey response for each article. If both the senior author and corresponding author respond to the survey, the corresponding author's responses will be used. Survey respondents will be asked if they consent to be contacted to participate in a subsequent interview. We will balance the sample among those that indicated interest in an interview based on level of knowledge about implementation science (a self-reported item to be included in the survey) and setting to ensure a wide range of user experiences are represented. We anticipate interviewing 20–30 users; an exact number will be determined by achievement of “meaning saturation” (25). A follow-up email will be sent to respondents who indicated interest in participating in an interview to arrange a suitable time for a video conference. Survey respondents will receive an honorarium for participation.

### 2.12 Survey item development

We will ask respondents to complete the System Usability Scale (SUS) (26, 27)—a simple, 10-item scale on the global view of IRLM usability. SUS items will be adapted to refer specifically to IRLM use. Other survey items will be informed by the results of the scoping review and citation analysis.

### 2.13 Semi-structured interview guide

The semi-structured interview guide will include topics of IRLM ease of use and challenges, with probing questions. We will also ask about types of training or assistance the participant received, or trainings that they believe would facilitate successful use of the IRLM.

### 2.14 Data analysis

Responses to the survey questions will be analyzed using descriptive statistics. We will generate a single number representing a composite measure of the overall usability of the IRLM following SUS scoring guidelines, with the score ranging from 0 to 100 and higher score indicating higher usability. Interviews will be recorded, transcribed, and analyzed thematically (25), paying attention to how narratives vary by participant type and setting, the version of IRLM used, and the purpose of use (e.g., implementation research, implementation practice, grant application).

### 2.15 Expert panel

We will form an expert panel with a subsample of IRLM users, implementation scientists, and other interested parties who are familiar with the IRLM to discuss overall feedback and recommendations for improving the IRLM. Participants will be invited through word of mouth to participate in a panel (similar to a focus group) based on their experience or familiarity with the IRLM. Panel meetings will be recorded, transcribed, and analyzed thematically with double coding and discrepancies resolved via consensus among the coders. Panel members will receive an honorarium for participation.

## 3 Discussion

The use of logic modeling is an important component to successfully planning and executing studies, particularly in implementation research. This scoping review and citation search will provide rich detail into how the IRLM has been used across service settings, which will allow the developers to improve the design of the IRLM as well as provide additional user guidance. The user survey and expert pan will provide additional feedback and also inform timely improvements to the design and function of the IRLM.

## 4 Dissemination

Results from these findings will be disseminated through peer-reviewed publication and conference presentations. As reviews are being finalized, searches will be updated to ensure that results include the current state of the literature. We will also use the findings from this study to inform and refine the free, interactive online IRLM tool, located at https://irlm.isgmh.northwestern.edu.

## References

[1] Merle JL Smith JD. Refining the implementation research logic model: a citation analysis and scoping review protocol. OSF Registry of the Protocol. (2024). 10.17605/OSF.IO/Y94BJ PMC11540644 39512272

[2] Nilsen P Bernhardsson S. Context matters in implementation science: a scoping review of determinant frameworks that describe contextual determinants for implementation outcomes. BMC Health Serv Res. (2019) 19(1):189. 10.1186/s12913-019-4015-3 30909897 PMC6432749

[3] Powell BJ Waltz TJ Chinman MJ Damschroder LJ Smith JL Matthieu MM A refined compilation of implementation strategies: results from the expert recommendations for implementing change (ERIC) project. Implement Sci. (2015) 10:21. 10.1186/s13012-015-0209-1 25889199 PMC4328074

[4] Lengnick-Hall R Williams NJ Ehrhart MG Willging CE Bunger AC Beidas RS Eight characteristics of rigorous multilevel implementation research: a step-by-step guide. Implement Sci. (2023) 18(1):52. 10.1186/s13012-023-01302-2 37872618 PMC10594828

[5] Lewis CC Klasnja P Powell BJ Lyon AR Tuzzio L Jones S From classification to causality: advancing understanding of mechanisms of change in implementation science. Front Public Health. (2018) 6:136. 10.3389/fpubh.2018.00136 29868544 PMC5949843

[6] Smith JD Li DH Rafferty MR. The implementation research logic model: a method for planning, executing, reporting, and synthesizing implementation projects. Implement Sci. (2020) 15(1). 10.1186/s13012-020-01041-8 32988389 PMC7523057

[7] Glasgow RE Harden SM Gaglio B Rabin B Smith ML Porter GC RE-AIM planning and evaluation framework: adapting to new science and practice with a 20-year review. Front Public Health. (2019) 7:64. 10.3389/fpubh.2019.00064 30984733 PMC6450067

[8] Dzewaltowski DA Glasgow RE Klesges LM Estabrooks PA Brock E. RE-AIM: evidence-based standards and a web resource to improve translation of research into practice. Ann Behav Med. (2004) 28(2):75–80. 10.1207/s15324796abm2802_1 15454353

[9] Klesges LM Estabrooks PA Dzewaltowski DA Bull SS Glasgow RE. Beginning with the application in mind: designing and planning health behavior change interventions to enhance dissemination. Ann Behav Med. (2005) 29(2):66–75. 10.1207/s15324796abm2902s_10 15921491

[10] Moullin JC Dickson KS Stadnick NA Rabin B Aarons GA. Systematic review of the exploration, preparation, implementation, sustainment (EPIS) framework. Implement Sci. (2019) 14(1):1. 10.1186/s13012-018-0842-6 30611302 PMC6321673

[11] Damschroder L Reardon CM Widerquist MAO Lowery JC. The updated consolidated framework for implementation research based on user feedback. Implement Sci. (2022) 17:75. 10.1186/s13012-022-01245-0 36309746 PMC9617234

[12] Damschroder LJ Reardon CM Opra Widerquist MA Lowery J. Conceptualizing outcomes for use with the consolidated framework for implementation research (CFIR): the CFIR outcomes addendum. Implement Sci. (2022) 17(1):7. 10.1186/s13012-021-01181-5 35065675 PMC8783408

[13] Hirt JA-O Nordhausen T Appenzeller-Herzog CA-OX Ewald HA-O. Using citation tracking for systematic literature searching - study protocol for a scoping review of methodological studies and a delphi study. F1000Res. (2021) 9:1386. (2046-1402 (Electronic)). 10.12688/f1000research.27337.3 34631036 PMC8474097

[14] Ding Y Zhang G Chambers T Song M Wang X Zhai C. Content-based citation analysis: the next generation of citation analysis. J Assoc Inf Sci Technol. (2014) 65(9):1820–33. 10.1002/asi.23256

[15] Peters MDJ Godfrey C McInerney P Khalil H Larsen P Marnie C Best practice guidance and reporting items for the development of scoping review protocols. JBI Evid Synth. (2022) 20(4):953–68. 10.11124/JBIES-21-00242 35102103

[16] Peters MDJ Godfrey C McInerney P Munn Z Tricco AC Khalil H. Chapter 11: Scoping Reviews (2020 version). In: Aromataris E Munn Z, editors. JBI Manual for Evidence Synthesis. JBI (2020). 10.46658/JBIMES-20-12

[17] Arksey H O'Malley L. Scoping studies: towards a methodological framework. Int J Soc Res Methodol. (2005) 8(1):19–32. 10.1080/1364557032000119616

[18] Tricco AC Lillie E Zarin W O'Brien KK Colquhoun H Levac D PRISMA extension for scoping reviews (PRISMA-ScR): checklist and explanation. Ann Intern Med. (2018) 169(7):467–73. 10.7326/M18-0850 30178033

[19] Rethlefsen ML Kirtley S Waffenschmidt S Ayala AP Moher D Page MJ PRISMA-S: an extension to the PRISMA statement for reporting literature searches in systematic reviews. Syst Rev. (2021) 10(1):39. 10.1186/s13643-020-01542-z 33499930 PMC7839230

[20] Smith J, editor. An implementation research logic model: a step toward improving scientific rigor, transparency, reproducibility, and specification. 11th Annual Conference on the Science of Dissemination and Implementation; AcademyHealth (2018).

[21] McGowan J Sampson M Salzwedel DM Cogo E Foerster V Lefebvre C. PRESS peer review of electronic search strategies: 2015 guideline statement. J Clin Epidemiol. (2016) 75:40–6. 10.1016/j.jclinepi.2016.01.021 27005575

[22] Godin K Stapleton J Kirkpatrick SI Hanning RM Leatherdale ST. Applying systematic review search methods to the grey literature: a case study examining guidelines for school-based breakfast programs in Canada. Syst Rev. (2015) 4:138. 10.1186/s13643-015-0125-0 26494010 PMC4619264

[23] Smith JD Li DH Hirschhorn LR Gallo C McNulty M Phillips G Landscape of HIV implementation research funded by the national institutes of health: a mapping review of project abstracts. AIDS Behav. (2020) 24(6):1903–11. 10.1007/s10461-019-02764-6 31845078 PMC7220870

[24] Covidence Systematic Review Software. Melbourne, Australia: Veritas Health Innovation (2022). Available online at: https://www.covidence.org

[25] Braun V Clarke V. Thematic analysis. In: Cooper H Camic PM Long DL Panter AT Rindskopf D Sher KJ, editors. APA Handbook of Research Methods in Psychology, Vol. 2. Research Designs: Quantitative, Qualitative, Neuropsychological, and Biological. Washington, DC: American Psychological Association (2012). p. 57–71. 10.1037/13620-004

[26] Sauro J. A Practical Guide to the System Usability Scale: Background, Benchmarks & Best Practices. Measuring Usability LLC (2011). Available online at: https://books.google.com/books?id=BL0kKQEACAAJ (accessed July 19, 2024).

[27] Suaro J. Measuring Usability with the System Usability Scale (SUS) (2011). Available online at: https://measuringu.com/sus/ (accessed July 19, 2024).

