# Supplementary material for: Refining the implementation research logic model: a citation analysis, user survey, and scoping review protocol
Source: Front Health Serv. 2024 Oct 24;4:1490764. doi: 10.3389/frhs.2024.1490764 (PMC11540644; doi:10.3389/frhs.2024.1490764)
Supplement: Supplementary file 1 [file Table1.docx]

**13 Supplemental Material**

Study Codebook with operational definition and levels

| **Code** | **Definition** | **Levels** |
| --- | --- | --- |
| author | First author last name | open text |
| year | Publication year | open text |
| journal | Journal published in | open text |
| title | Article title | open text |
| Research Item Type |  | journal article, conference presentation, poster, other |
| If other research type: what? |  | open text |
| Included some logic model | Authors included some logic model (not necessarily the IRLM) | yes/no |
| included IRLM image? | authors included a unique IRLM with data | yes/no |
| Authors indicate they created an IRLM but did not include it in manuscript |  | yes/no |
| Study Setting (larger level and sub-level) | where the study took place | educational context, healthcare related (primary care, health system/non-primary care), community setting, multi-sector (multi select option). |
| Study Setting (verbatim) | Indicate verbatim what the authors state the study setting is | open text |
| Evidence-based program being Implemented | What is the evidence-based program, practice, policy being implemented? | open text |
| Primary outcome of program being implemented | What is the primary outcome of the “thing” being implemented? | health outcome, mental health outcome, physical health outcome, health behavior outcome, educational outcome |
| Method of data collection for IRLM (select all that apply): | How data was collected to populate the IRLM | interviews, focus groups, survey, literature review, other |
| if other method, include |  | open text |
| Engaged community partners/stakeholders with IRLM? | Did the study team or authors engage their community/implementation partners in the development or refinement of the IRLM | yes/no |
| if yes, what level of involvement with CE? |  | Outreach, Consult, Involve, Collaborate, Shared Leadership. |
| Location of IRLM Image | where in the manuscript was the IRLM image included? | Figure in main article, supplement, linked to external source. |
| Version or Template used | which version/template of IRLM did authors use? (see Smith et al., 2020 for versions) | Standard Form (no intervention); Standard Form (with intervention); Comparative Intervention; Implementation of an Intervention Across or Linking 2 Contexts; Implementation Optimization Study or SMART/adaptive intervention; Adjunctive Intervention; Customized: |
| if customized version, how so? / other unique features | any customizations or unique elements added to the IRLM | open text |
| COSMOS linking? | to what extent did users complete linking of determinants-strategies-mechanisms-outcomes? Comprehensive means all COSMOS. | none, partial, comprehensive |
| if partial linking, specify |  | determinants-strategies,  strategies-outcomes,  determinants-outcomes,  mechanisms-outcomes,  other |
| other partial linking specify | if none of the four listed, what | open text |
| linking method (multi-select) |  | subscripts superscripts color coding font effects: bolding/italicizing/Underlining other: describe |
| If other linking method; describe |  | open text |
| included Determinant Valence? | did authors use +/- to determine barriers and facilitators? | yes/no |
| Indicate Primary Outcome in the figure? | did the authors indicate primary outcome(s) in their IRLM figure in some way? | yes/no |
| Determinant Framework | what determinant framework was used to fill out IRLM? | Select multiple:  CFIR 1.0 vs CFIR 2.0 EPIS PRISM I-Parhis/Parhis  ISF Other  No framework |
| if other, what |  | open text |
| Strategy Framework | what strategy framework was used to fill out IRLM? | Select multiple:  ERIC EPOCH Behavior Change Wheel/ TDF/COM-B Jennifer Leeman et al., taxonomy Balis, Harden et al community strategies  Cook et al school strategies (SISTER)  Other: No framework |
| if other, what |  | open text |
| Outcome Framework | what outcome framework was used to fill out the IRLM. | Select multiple:  RE-AIM IOF  PIPE Other  No framework |
| if other, what |  | open text |
| Specify other theory, model, or framework not already reported? | needed to be used to inform IRLM | open text |
| Included Mechanisms? | Code yes/no if authors included mechanisms as part of their IRLM | Yes/No |
| If yes, coding approach to mechanisms | If authors included mechanisms, what approach did they use | Select one:  Process  Event  Mediator  Other  unspecified |
| If yes, Mechanism Source | If authors included mechanisms, from what source did they identify them | Theory  Empirical evidence  Other (open text) |
| If yes, copy/paste verbatim stated mechanisms |  | Open text |
| If yes, Mechanism Framework and/or Theory | If authors included mechanisms, what framework or theory did they use? | Open text |
| Specify any other theory, model, or framework not already reported? | *needed to be used to inform IRLM | open text |
| Stage of research when IRLM was used (select all that apply: Planning, Reporting, Executing, Synthesizing ) | stages of change as reported in IRLM paper (Smith, 2020) | Planning, Reporting, Executing, Synthesizing |
| Benefits of IRLM | reported benefits of using the IRLM for their project | Open text |
| Noted deficiencies - anything that's unclear (read discussion section carefully) | noted deficiencies related to the IRLM specifically noted by authors | Y/N; Open text |
| Recommendations for improving any aspect of IRLM |  | Y/N; Open text |
| For included articles that don’t have a unique IRLM figure/table, start here | | |
| Citation Purpose (e.g., discussing theory? Logic models in general?) |  | Open text |
| Other logic models included | If authors use or cite other logic models, indicate their name/citation | Y/N; Open text |
| What section of the paper was the citation used? (include all that apply) |  | Introduction, method, results, discussion |
| Benefits of IRLM | Report any noted benefits the authors state to using the IRLM | Open text |
| Noted deficiencies? | Report any noted deficiencies related to the IRLM | Open text |
| Recommendations for improving any aspect of IRLM | Report any recommendations the authors make for improving the IRLM | Open text |
