# Supplementary material for: Refining the implementation research logic model: a citation analysis, user survey, and scoping review protocol
Source: Front Health Serv. 2024 Oct 24;4:1490764. doi: 10.3389/frhs.2024.1490764 (PMC11540644; doi:10.3389/frhs.2024.1490764)
Supplement: Supplementary file 2 [file Table2.docx]

**13 Supplemental Material**

Database Search Strategies: Implementation Research Logic Model Scoping Review

Databases include Medline (Ovid) 1946-2024, Embase (Elsevier) 1974 - 2024, CINAHL Complete (Ebscohost) 1937-2024, Cochrane Library (Wiley) 1898 - 2024 including CENTRAL (wiley.com) 1898-2024, APA PsycINFO (Ebscohost) 1872-2024, ProQuest Dissertations & Theses Global 1861-2024, Scopus (Elsevier) 1970-2024 and Web of Science Core Collection (Clarivate) 1900-2024. A date limit from 2018 will be applied.

September 19, 2024 – all databases ran and results exported.

**Search concepts table for Ovid Medline:**

| Search Statements | Concepts | Term fields |
| --- | --- | --- |
| #1 - #6 | IRLM | keywords |
| #7 and #8 | IR + Logic Model,LM | keywords |
| #11 and (#14-#16) | IR + (Logic set or Models subject)) | keyword, subject terms |
| #11 and #14 #18 | IR + Logic set + Models set | keyword, subject terms |
| (and/11,19) and (#8 or #17) | IR + (Research Design subject) + (LM keywords or model keywords) | keyword, subject terms |
| #11 and #19 and #20 | IR + Research Design-subject + DeliveryOfHC-subject | keyword, subject terms |

Abbreviations: IRLM = Implementation Research Logic Model

IR = Implementation Research

LM = Logic Model

DeliveryOfHC = Delivery of Health Care [MeSH term]

**Medline (Ovid)**

September 19, 2024

Ovid MEDLINE(R) and Epub Ahead of Print, In-Process, In-Data-Review & Other Non-Indexed Citations, Daily and Versions <1946 to September 18, 2024>

1 ("Implementation Research Logic Model*" or IRLM).ti,ab,kf,kw. [IRLM keywords 1] 44

2 ((Implementation or pre-implementation) and "logic model*").ti,ab,kf. [IRLM keywords 2] 550

3 (((Implementation or pre-implementation) adj2 (research or science* or scientific)) and "logic model*").ti,ab,kf,kw. [IRLM keywords 3] 101

4 ((Implementation or pre-implementation) adj2 (research or science* or scientific) adj6 (logic or model*)).ti,ab,kf,kw. [IRLM keywords 4] 485

5 ((Implementation or pre-implementation) adj2 (research or science* or scientific) adj2 (logic or model* or process* or strategy or strategies)).ti,ab,kf,kw. [No framework - IRLM keywords 5 ] 420

6 ((Implementation or pre-implementation) adj2 (research or science* or scientific)).ti,ab,kf,kw. and ("implementation mapping".ti,ab,kf,kw. or ("implementation strateg*" or innovation*).ti.) [IRLM keywords 6] 460

7 ((Implementation or pre-implementation) adj2 (research or science* or scientific)).ti,ab,kf,kw. [IR keyword terms] 13657

8 ("logic model*" or "logic map*" or "concept map*" or "concept* model*" or "graphic* depiction*" or "graphic* representation*" or (mapping adj2 model*) or "road map*").ti,ab,kf,kw. [LM keyword synonyms] 23839

9 implementation science/ [ IR subject term] 1543

10 ("implementation science" or "implementation research" or "implementation scien* research").ti,ab,kf,kw. [IR keywords] 10781

11 or/9-10 [IR set] 11208

12 logic/ [Logic subject term] 4349

13 (logic or logics).ti,ab,kf,kw. [Logic keyword] 26728

14 or/12-13 [Logic set] 29034

15 exp Models, Theoretical/ [theoretical models subject term] 1999978

16 exp models, statistical/ or logistic models/ [statistical models subject terms] 461777

17 (model or models or modeling or modelling).ti,ab,kf,kw. [model keywords] 4094130

18 or/15-17 [Model set] 5111420

19 Research Design/ [subject term] 129183

20 "Delivery of Health Care"/ [subject term ] 123651

21 ("36522303" or "36514111" or "31275915" or "30353149").ui. [4 Exemplars] 4

22 or/1-6 [IRLM keyword sets] 1645

23 and/7-8 [IR + LM keyword terms set] 272

24 11 and (or/14-16) [IR + (Logic / Models subject) set] 371

25 and/11,14,18 [IR + Logic + Models set] 102

26 (and/11,19) and (or/8,17) [IR + (Research Design subject) + (LM or model) set] 130

27 and/11,19-20 [IR + Research Design-subject + DeliveryOfHC-subject set] 59

28 or/22-27 [Final set 1] 2103

29 limit 28 to yr="2018 - 2024" [Final set 1] 1531

30 or/21,29 [Final set 1 finds exemplar] **1531**

Medline (Ovid) legend:

Field codes:

/ = Medical Subject Heading (MeSH); ti = article title; ab = abstract; kf = keyword heading word; kw = keyword heading (author keywords); ui = unique identifier

Proximity operator: adj#

Truncation:  *

**Embase (Elsevier)**

September 19, 2024

#31 #29 AND 'conference abstract'/it 266

#30 #29 NOT 'conference abstract'/it 1,675

#29 (#21 OR #22 OR #23 OR #25 OR #26 OR #27) AND [2018-2024]/py **1,941**

#28 #21 OR #22 OR #23 OR #25 OR #26 OR #27 2,668

#27 #11 AND #19 AND #20 58

#26 #11 AND (#8 OR #17) AND #19 126

#25 #11 AND #14 AND #18 99

#24 #11 AND #14 AND (#15 OR #16) 10

#23 #11 AND (#14 OR #15 OR #16) 875

#22 #7 AND #8 250

#21 #1 OR #2 OR #3 OR #4 OR #5 OR #6 1,707

#20 'health care delivery'/de 219,610

#19 'methodology'/de 1,679,992

#18 #15 OR #16 OR #17 5,618,005

#17 model:ti,ab,kw OR models:ti,ab,kw OR modeling:ti,ab,kw OR modelling:ti,ab,kw 5,148,885

#16 'statistical model'/exp 744,636

#15 'theoretical model'/exp 152,729

#14 #12 OR #13 33,533

#13 logic:ti,ab,kw OR logics:ti,ab,kw 30,556

#12 'logic'/de OR 'logical reasoning'/de 9,721

#11 #9 OR #10 13,693

#10 'implementation science':ti,ab,kw OR 'implementation research':ti,ab,kw OR 'implementation scien* research':ti,ab,kw 11,205

#9 'implementation science'/de 6,567

#8 'logic model*':ti,ab,kw OR 'logic map*':ti,ab,kw OR 'concept map*':ti,ab,kw OR 'concept* model*':ti,ab,kw OR 'graphic* depiction*':ti,ab,kw OR 'graphic* representation*':ti,ab,kw OR ((mapping NEAR/2 model*):ti,ab,kw) OR 'road map*':ti,ab,kw 27,693

#7 ((implementation OR 'pre implementation') NEAR/2 (research OR science* OR scientific)):ti,ab,kw 14,639

#6 (((implementation OR 'pre implementation') NEAR/2 (research OR science* OR scientific)):ti,ab,kw) AND ('implementation mapping':ti,ab,kw OR 'implementation strateg*':ti OR innovation*:ti) 423

#5 ((implementation OR 'pre implementation') NEAR/2 (research OR science* OR scientific) NEAR/2 (logic OR model* OR process* OR strategy OR strategies)):ti,ab,kw 372

#4 ((implementation OR 'pre implementation') NEAR/2 (research OR science* OR scientific) NEAR/6 (logic OR model*)):ti,ab,kw 504

#3 (((implementation OR 'pre implementation') NEAR/2 (research OR science* OR scientific)):ti,ab,kw) AND 'logic model*':ti,ab,kw 88

#2 (implementation:ti,ab,kw OR 'pre implementation':ti,ab,kw) AND 'logic model*':ti,ab,kw 627

#1 'implementation research logic model*':ti,ab,kw OR irlm:ti,ab,kw 43

Embase legend:

Field codes: ti = article Title, ab = Abstract, kw = Keyword, de = Index (descriptor) term ), mj = Focused (Descriptor)Index term

Proximity operator: NEAR/#

Truncation:  *

**CINAHL Complete (EBSCOhost)**

September 19, 2024

# Query Limiters/Expanders Results

S26 S25 Limiters - Publication Date: 20180101-20241031 **987**

S25 S20 OR S21 OR S22 OR S23 OR S24 1,519

S24 S11 AND S18 AND S19 27

S23 S11 AND S18 AND (S8 OR S16) 48

S22 S11 AND S14 AND S17 36

S21 S7 AND S8 111

S20 S1 OR S2 OR S3 OR S4 OR S5 OR S6 1,443

S19 (MH "Health Care Delivery") 69,463

S18 (MH "Study Design") 37,644

S17 S15 OR S16 746,444

S16 TI ( (model or models or modeling or modelling) ) OR AB ( (model or models or modeling or modelling) ) 657,754

S15 (MH "Models, Theoretical+") OR (MH "Models, Statistical+") 204,790

S14 S12 OR S13 5,655

S13 TI ( (logic or logics) ) OR AB ( (logic or logics) ) 4,759

S12 (MH "Logic") 1,382

S11 S9 OR S10 4,754

S10 TI ( ("implementation science" or "implementation research" or "implementation scien* research") ) OR AB ( ("implementation science" or "implementation research" or "implementation scien* research") ) 3,840

S9 (MH "Implementation Science") 1,515

S8 TI ( ("logic model*" or "logic map*" or "concept map*" or "concept* model*" or "graphic* depiction*" or "graphic* representation*" or (mapping N2 model*) or "road map*") ) OR AB ( ("logic model*" or "logic map*" or "concept map*" or "concept* model*" or "graphic* depiction*" or "graphic* representation*" or (mapping N2 model*) or "road map*") ) 9,878

S7 TI ( ((Implementation or pre-implementation) N2 (research or science* or scientific)) ) OR AB ( ((Implementation or pre-implementation) N2 (research or science* or scientific)) ) 6,497

S6 TI ( ((Implementation or pre-implementation) N2 (research or science* or scientific)) AND ("implementation mapping" or ("implementation strateg*" or innovation*) ) OR AB ( ((Implementation or pre-implementation) N2 (research or science* or scientific)) AND ("implementation mapping" or ("implementation strateg*") ) 764

S5 TI ( ((Implementation or pre-implementation) N2 (research or science* or scientific) N2 (logic or model* or process* or strategy or strategies)) ) OR AB ( ((Implementation or pre-implementation) N2 (research or science* or scientific) N2 (logic or model* or process* or strategy or strategies)) ) 417

S4 TI ( ((Implementation or pre-implementation) N2 (research or science* or scientific) N6 (logic or model*)) ) OR ( ((Implementation or pre-implementation) N2 (research or science* or scientific) N6 (logic or model*)) ) 297

S3 TI ( (((Implementation or pre-implementation) N2 (research or science* or scientific)) and "logic model*") ) OR AB ( (((Implementation or pre-implementation) N2 (research or science* or scientific)) and "logic model*") ) 33

S2 TI ( ((Implementation or pre-implementation) and "logic model*") ) OR AB ( ((Implementation or pre-implementation) and "logic model*") ) 282

S1 TI ( ("Implementation Research Logic Model*" or IRLM) ) OR AB ( ("Implementation Research Logic Model*" or IRLM) ) 13

CINAHL legend:

Field codes: TI = title, AB= abstract, MH = CINAHL Exact Subject Headings, MJ = CINAHL Word in Major Subject Heading, MW = CINAHL Heading Word

Proximity operator: N#

Truncation:  *

**Cochrane Library (Wiley)** September 19, 2024

- Cochrane Database of Systematic Reviews (CDSR)

- Cochrane Central Register of Controlled Trials (CENTRAL)

ID Search Hits

#1 (("Implementation Research Logic Model*" or IRLM)):ti,ab,kw 5

#2 (((Implementation or pre-implementation) and "logic model*")):ti,ab,kw 79

#3 ((((Implementation or pre-implementation) NEAR/2 (research or science* or scientific)) and "logic model*")):ti,ab,kw 11

#4 (((Implementation or pre-implementation) NEAR/2 (research or science* or scientific) NEAR/6 (logic or model*))):ti,ab,kw 54

#5 (((Implementation or pre-implementation) NEAR/2 (research or science* or scientific) NEAR/2 (logic or model* or process* or strategy or strategies))):ti,ab,kw 68

#6 (((Implementation or pre-implementation) NEAR/2 (research or science* or scientific)) AND ("implementation mapping" OR "implementation strateg*" or innovation*)):ti,ab,kw 93

#7 (((Implementation or pre-implementation) NEAR/2 (research or science* or scientific))):ti,ab,kw 1675

#8 (("logic model*" or "logic map*" or "concept map*" or "concept* model*" or "graphic* depiction*" or "graphic* representation*" or (mapping NEAR/2 model*) or "road map*")):ti,ab,kw 395

#9 MeSH descriptor: [Implementation Science] this term only 106

#10 (("implementation science" or "implementation research" or "implementation scien* research")):ti,ab,kw 1390

#11 #9 OR #10 1390

#12 MeSH descriptor: [Logic] explode all trees 126

#13 ((logic or logics)):ti,ab,kw 909

#14 #12 or #13 909

#15 MeSH descriptor: [Models, Theoretical] explode all trees 31204

#16 MeSH descriptor: [Models, Statistical] explode all trees 23150

#17 ((model or models or modeling or modelling)):ti,ab,kw 186357

#18 #15 or #16 or #17 186903

#19 MeSH descriptor: [Research Design] this term only 15905

#20 MeSH descriptor: [Delivery of Health Care] this term only 1629

#22 #1 or #2 or #3 or #4 or #5 or #6 257

#23 #7 and #8 15

#24 #11 and (#14 or #15 or #16) 30

#25 #11 and #14 and #18 13

#26 #11 and #19 and (#8 or #17) 29

#27 #11 and #19 and #20 4

#28 #22 or #23 or #24 of #25 or #26 or #27 285

#29 #28 with Publication Year from 2018 to 2024, in Trials 213

#30 #29 i**n Trials 213**

#31 #29 in Cochrane Reviews 0

Cochrane Library legend:

Field codes: ti = Title, ab = Abstract, kw = Keyword, MeSH descriptor = Medical Subject Heading

Proximity operator: NEAR/#

Truncation: *

**APA PsycInfo (EBSCOhost)**

Search History September 19, 2024

# Query Limiters/Expanders Results

S16 S15 Limiters - Publication Year: 2018-2024 **672**

S15 S12 OR S13 OR S14 1,176

S14 S9 AND (S8 OR (S10 AND S11)) 65

S13 S7 AND S8 103

S12 S1 OR S2 OR S3 OR S4 OR S5 OR S6 1,120

S11 TI ( (model or models or modeling or modelling) ) OR AB ( (model or models or modeling or modelling) ) OR KW ( (model or models or modeling or modelling) ) 928,883

S10 TI ( (logic or logics) ) OR AB ( (logic or logics) ) OR KW ( (logic or logics) ) 23,476

S9 TI ( ("implementation science" or "implementation research" or "implementation scien* research") ) OR AB ( ("implementation science" or "implementation research" or "implementation scien* research") ) OR KW ( ("implementation science" or "implementation research" or "implementation scien* research") ) 3,011

S8 TI ( ("logic model*" or "logic map*" or "concept map*" or "concept* model*" or "graphic* depiction*" or "graphic* representation*" or (mapping N2 model*) or "road map*") ) OR AB ( ("logic model*" or "logic map*" or "concept map*" or "concept* model*" or "graphic* depiction*" or "graphic* representation*" or (mapping N2 model*) or "road map*") ) OR KW ( ("logic model*" or "logic map*" or "concept map*" or "concept* model*" or "graphic* depiction*" or "graphic* representation*" or (mapping N2 model*) or "road map*") ) 19,934

S7 TI ( ((Implementation or pre-implementation) N2 (research or science* or scientific)) ) OR AB ( ((Implementation or pre-implementation) N2 (research or science* or scientific)) ) OR KW ( ((Implementation or pre-implementation) N2 (research or science* or scientific)) ) 6,185

S6 TI ( ((Implementation or pre-implementation) N2 (research or science* or scientific)) AND ("implementation mapping" or ("implementation strateg*" or innovation*) ) OR AB ( ((Implementation or pre-implementation) N2 (research or science* or scientific)) AND ("implementation mapping" or ("implementation strateg*") ) OR KW ( ((Implementation or pre-implementation) N2 (research or science* or scientific)) AND ("implementation mapping" or ("implementation strateg*") ) 491

S5 TI ( ((Implementation or pre-implementation) N2 (research or science* or scientific) N2 (logic or model* or process* or strategy or strategies)) ) OR AB ( ((Implementation or pre-implementation) N2 (research or science* or scientific) N2 (logic or model* or process* or strategy or strategies)) ) OR KW ( ((Implementation or pre-implementation) N2 (research or science* or scientific) N2 (logic or model* or process* or strategy or strategies)) ) 389

S4 TI ( ((Implementation or pre-implementation) N2 (research or science* or scientific) N6 (logic or model*)) ) OR ( ((Implementation or pre-implementation) N2 (research or science* or scientific) N6 (logic or model*)) ) OR KW ( ((Implementation or pre-implementation) N2 (research or science* or scientific) N6 (logic or model*)) ) 242

S3 TI ( (((Implementation or pre-implementation) N2 (research or science* or scientific)) and "logic model*") ) OR AB ( (((Implementation or pre-implementation) N2 (research or science* or scientific)) and "logic model*") ) OR KW ( (((Implementation or pre-implementation) N2 (research or science* or scientific)) and "logic model*") ) 18

S2 TI ( ((Implementation or pre-implementation) and "logic model*") ) OR AB ( ((Implementation or pre-implementation) and "logic model*") ) OR KW ( ((Implementation or pre-implementation) and "logic model*") ) 228

S1 TI ( ("Implementation Research Logic Model*" or IRLM) ) OR AB ( ("Implementation Research Logic Model*" or IRLM) ) OR KW ( ("Implementation Research Logic Model*" or IRLM) ) 6

APA PsycInfo legend:

Field codes: TI = title, AB = abstract,  KW = keywords, DE = Subject term

Proximity operator: N#

Truncation:  *

**ProQuest Dissertations & Theses Global (ProQuest)**

September 19, 2024

S6 [S2] OR [S3] OR [S4] OR [S5]

Limits applied: pd(20180101-20241231) **298**

S5 [S2] OR [S3] OR [S4] OR [S5] 1,467

S4 noft(("implementation science" or "implementation research" or "implementation scien* research")) AND noft((logic or logics)) AND (model or models or modeling or modelling) 5

S3 noft(((Implementation or pre-implementation) NEAR/2 (research or science* or scientific))) AND noft(("logic model*" or "logic map*" or "concept map*" or "concept* model*" or "graphic* depiction*" or "graphic* representation*" or (mapping NEAR/2 model*) or "road map*")) 142

S2 noft(((Implementation OR pre-implementation) NEAR/2 (research OR science* OR scientific)) ) AND title(("implementation mapping" OR "implementation strateg*" OR innovation*)) 102

S1 noft(("Implementation Research Logic Model*" OR IRLM)) OR noft(((Implementation OR pre-implementation) AND "logic model*")) OR noft(((Implementation OR pre-implementation) NEAR/2 (research OR science* OR scientific) NEAR/6 (logic OR model*))) OR noft(((Implementation OR pre-implementation) NEAR/2 (research OR science* OR scientific) NEAR/2 (logic OR model* OR process* OR strategy OR strategies))) 1,265

ProQuest Dissertations & Theses Global legend:

Field codes: NOFT = Anywhere except full text (NOFT) searches the full bibliographic record, but does NOT include a search of the full text.

Proximity operator: NEAR/#

Truncation:  *

**Scopus (Elsevier)**

September 19, 2024

( ( TITLE-ABS-KEY ( ( "Implementation Research Logic Model*" OR irlm ) ) OR TITLE-ABS-KEY ( ( ( implementation OR pre-implementation ) AND "logic model*" ) ) OR TITLE-ABS-KEY ( ( ( ( implementation OR pre-implementation ) W/2 ( research OR science* OR scientific ) ) AND "logic model*" ) ) OR TITLE-ABS-KEY ( ( ( "implementation science" OR "implementation research" OR "implementation scien* research" ) W/6 ( logic OR model* ) ) ) OR TITLE-ABS-KEY ( ( ( "implementation science" OR "implementation research" OR "implementation scien* research" ) W/2 ( logic OR model* OR process* OR strategy OR strategies ) ) ) ) ) OR ( ( TITLE-ABS-KEY ( ( "implementation science" OR "implementation research" OR "implementation scien* research" ) ) AND TITLE-ABS-KEY ( ( "logic model*" OR "logic map*" OR "concept map*" OR "concept* model*" OR "graphic* depiction*" OR "graphic* representation*" OR ( mapping W/2 model* ) OR "road map*" ) ) ) ) OR ( ( TITLE-ABS-KEY ( ( "implementation science" OR "implementation research" OR "implementation scien* research" ) ) AND ( TITLE-ABS-KEY ( ( "logic model*" OR "logic map*" OR "concept map*" OR "concept* model*" OR "graphic* depiction*" OR "graphic* representation*" OR ( mapping W/2 model* ) OR "road map*" ) ) ) OR TITLE ( model OR models OR modeling OR modelling ) ) ) OR ( ( TITLE-ABS-KEY ( "implementation science" OR "implementation research" OR "implementation scien* research" ) AND TITLE ( "implementation mapping" OR "implementation strateg*" ) ) ) AND PUBYEAR > 2017 AND PUBYEAR < 2025

**1,922 results**

Scopus legend:

Field codes: TITLE-ABS-KEY = Document Title, Abstract, Keywords

Proximity operator: W/

Truncation: *

**Web of Science Core Collection (Clarivate)**

Science Citation Index Expanded (SCI-EXPANDED)

Social Sciences Citation Index (SSCI)

Arts & Humanities Citation Index (A&HCI)

Emerging Sources Citation Index (ESCI)

September 19, 2024

#7 Search: #1 OR #2 OR #3 OR #4 OR #5 Timespan: 2018-01-01 to 2024-10-01 Results: **2081**

#6 Search: #1 OR #2 OR #3 OR #4 OR #5 Results: 2934

#5 Search: ("implementation science" or "implementation research" or "implementation scien* research") (Topic) AND (logic or logics) (Topic) AND (model or models or modeling or modelling) (Topic) Results: 112

#5 Search: ((Implementation or pre-implementation) NEAR/2 (research or science* or scientific)) (Topic) AND ("logic model*" or "logic map*" or "concept map*" or "concept* model*" or "graphic* depiction*" or "graphic* representation*" or (mapping NEAR/2 model*) or "road map*") (Topic) Results: 361

#3 Search: ((Implementation or pre-implementation) NEAR/2 (research or science* or scientific)) (Topic) AND ("implementation strateg*" or innovation*) (Title) Results: 605

#2 Search: ((Implementation or pre-implementation) NEAR/2 (research or science* or scientific)) AND "implementation mapping" (Topic) Results: 39

#1 Search: ("Implementation Research Logic Model*" or IRLM) (Topic) OR ((Implementation or pre-implementation) and "logic model*") (Topic) OR ((Implementation or pre-implementation) NEAR/2 (research or science* or scientific) NEAR/6 (logic or model*)) (Topic) OR ((Implementation or pre-implementation) NEAR/2 (research or science* or scientific) NEAR/2 (logic or model* or process* or strategy or strategies)) (Topic) Results: 2196

Web of Science Core Collection legend:

Field codes: TS  = topic (Title, Abstract, Author Keywords, Keywords Plus®)

Proximity operator: NEAR/#

Truncation:  *
